# Isolimonic acid interferes with *Escherichia coli* O157:H7 biofilm and TTSS in QseBC and QseA dependent fashion

**DOI:** 10.1186/1471-2180-12-261

**Published:** 2012-11-15

**Authors:** Amit Vikram, Palmy R Jesudhasan, Suresh D Pillai, Bhimanagouda S Patil

**Affiliations:** 1 Vegetable and Fruit Improvement Center, Department of Horticultural Sciences, Texas A &M University, Texas, 77843-2119, USA; 2 Food Safety & Environmental Microbiology Program, Texas A&M University, College Station, College Station, Texas, 77843-2472, USA

**Keywords:** Quorum sensing, Natural products, Limonoids, Citrus, *Escherichia coli* O157:H7, LEE, Epinephrine

## Abstract

**Background:**

*E. coli* O157:H7 (EHEC) is an important human pathogen. The antibiotic treatment of EHEC reportedly results in release of Shiga toxin and is therefore discouraged. Consequently, alternative preventive or therapeutic strategies for EHEC are required. The objective of the current study was to investigate the effect of citrus limonoids on cell-cell signaling, biofilm formation and type III secretion system in EHEC.

**Results:**

Isolimonic acid and ichangin were the most potent inhibitors of EHEC biofilm (IC_25_=19.7 and 28.3 μM, respectively) and adhesion to Caco-2 cells. The qPCR analysis revealed that isolimonic acid and ichangin repressed LEE encoded genes by ≈3 to 12 fold. In addition, *flhDC* was repressed by the two limonoids (≈3 to 7 fold). Further studies suggested that isolimonic acid interferes with AI-3/epinephrine activated cell-cell signaling pathway. Loss of biofilm inhibitory activity of isolimonic acid in *ΔqseBC* mutant, which could be restored upon complementation, suggested a dependence on functional QseBC. Additionally, overexpression of *qseBC* in wild type EHEC abated the inhibitory effect of isolimonic acid. Furthermore, the isolimonic acid failed to differentially regulate *ler* in *ΔqseA* mutant, while plasmid borne expression of *qseA* in *ΔqseA* background restored the repressive effect of isolimonic acid.

**Conclusions:**

Altogether, results of study seem to suggest that isolimonic acid and ichangin are potent inhibitors of EHEC biofilm and TTSS. Furthermore, isolimonic acid appears to interfere with AI-3/epinephrine pathway in QseBC and QseA dependent fashion.

## Background

Enterohaemorrhagic *Escherichia coli* (EHEC) is a major foodborne pathogen associated with frequent outbreaks of diarrheal disease. Most individuals develop watery diarrhea and recover. However, about 15–20% cases may develop life-threatening bloody diarrhea and hemolytic uremic syndrome (HUS) [1,2]. Dissemination and contact of humans with EHEC from multiple sources such as undercooked meats, raw fruits and vegetables, physical contact with EHEC harboring animals further contribute to increased frequency of illness [2,3].

EHEC is usually ingested through contaminated food products. Once inside the host, EHEC traverses to colon and establishes itself in the distal ileum or large bowel. Inside the colon, EHEC is thought to use guided motility, provided by flagellar motion, to reach its preferred site of attachment [4]. Autoinducer molecules (AI-2/AI-3) and hormones (epinephrine/norepinephrine) induce various virulence factors and are speculated to help in attachment and subsequent infection process [5]. A two-component system QseBC [6] induces flagellar operon in response to hormones and AI-2/AI-3, resulting in increased and guided motility [4] towards epithelial cell layer. Upon encountering the epithelial cell layer, the flagella and other surface structures such as type 1 pili and hemorrhagic coli pilus help EHEC to attach to the surface [7-9]. Multiple environmental and genetic factors such as pH, hormones, signaling molecules as well as quorum sensing (QS) regulate the expression of Locus of enterocyte effacement (LEE) and flagellar operons [10-13]. The hormones and AI-3 also induce type III secretion system (TTSS) in EHEC through QseEF and QseAD [14,15]. TTSS is encoded in LEE, which is organized in five operons LEE1-LEE5. LEE1-encoded regulator (Ler) is the first gene on LEE1 operon and subject to modulation by various regulators. In turn, Ler activates the transcription of the five operons [13,15,16].

The TTSS penetrates the host cell membrane and serves as conduit for injecting effector proteins. These effector proteins manipulate the host machinery including actin cytoskeleton, resulting in attaching and effacing lesions. Some of the secreted effectors disrupt the tight junction leading to higher secretion of chloride ions and ultimately developing in diarrhea [17]. The phage encoded Shiga toxin is the main virulence factor of EHEC and other Shiga toxin producing *E. coli*. The Shiga toxin disrupts the protein synthesis in host epithelial cells causing necrosis and cell death [17]. Additionally, Shiga toxin travels to kidney through blood stream and damages renal endothelial cells inciting renal inflammation, potentially leading to HUS [2,18]. Along with the direct injury to epithelial cells, biofilms formed by pathogenic *E. coli* strains can pose serious health problems such as prostatitis, biliary tract infections, and urinary catheter cystitis [19].

Antibiotics and antidiarrheal drug therapy of EHEC activates the stress response resulting in induction of phage lytic cycle and subsequent release of Shiga toxin. The release of Shiga toxin is directly correlated with increase in HUS incidence [2,18]. At present, CDC recommends preventive measures such as washing hands and thorough cooking of meats etc. to control EHEC infections. However, these preventive measures need to be supported with alternative strategies for prevention and control of EHEC infections. A promising strategy is to identify anti-virulence agents, which may be used alone or in conjunction with antibiotic therapy [20]. Anti-virulence agents target bacterial virulence determinants including toxin production, adhesion to host cells, specialized secretion systems such as TTSS [21]. Application of anti-virulence agents is speculated to allow host immune system to prevent or clear the bacterial infection. Several synthetic and natural molecules with anti-virulence properties have been discovered [20,21] and at least one molecule, LED209, was shown to be effective in animal models [20]. However, none of the molecules have entered wide-scale clinical trial as of yet, owing to various concerns such as their toxicity and safety. Therefore, there is an urgent need to identify a more diverse pool of molecules with anti-virulence activities. Availability of such a pool will ensure better drug designing strategies, to combat bacterial infections like EHEC.

Secondary metabolites produced by plants present very diverse scaffolds, which have been used for designing novel drugs including antimicrobials. In nature, secondary metabolites contribute to systemic and induced plant defense system against insect, bacterial and fungal infestation [22]. Several secondary metabolites belonging to classes such as coumarins, flavonoids, terpenoids and alkaloids demonstrate inhibitory properties against numerous microorganisms. Recently our group and others identified QS inhibitory properties of several plant secondary metabolites and extracts rich in phytochemicals [23-28].

**Figure 1:**
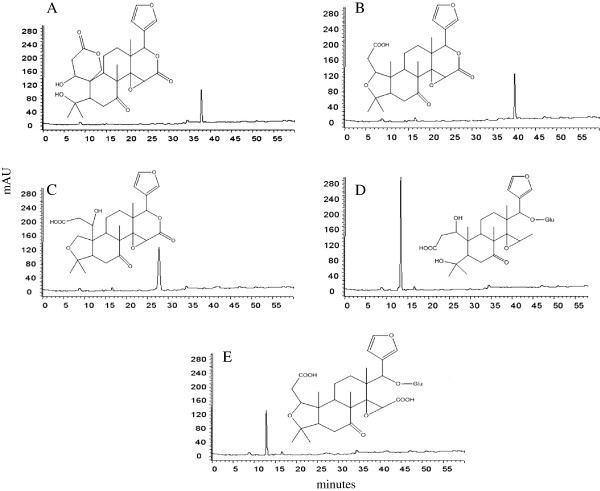
**HPLC chromatograms and structures of limonoids.** The limonoids were analyzed using HPLC. Purity was determined by calculating percentage area under curve for the given limonoids. The figure depicts chromatogram and structure of (**A**) ichangin, (**B**) isoobacunoic acid, (**C**) isolimonic acid, (**D**) DNAG, (**E**) IOAG.

Citrus species contain a unique class of secondary metabolites known as limonoids. Chemically, limonoids are triterpenoids with relatively high degree of oxygenation [29]. Several studies have reported anticancer, cholesterol lowering, antiviral and antifeedant activities of citrus limonoids [29-35]. Recently, we demonstrated that certain limonoids such as obacunone, nomilin, isolimonic acid and ichangin interfere with QS in *V. harveyi*[23,36]. In addition, obacunone and nomilin seems to modulate type III secretion system (TTSS) and biofilm formation in EHEC and *Salmonella* Typhimurium [23,37]. The present work was carried out to understand effect of five citrus limonoids (Figure 1), *viz.* isolimonic acid, ichangin, isoobacunoic acid, isoobacunoic acid glucoside (IOAG) and deacetyl nomilinic acid glucoside (DNAG) on EHEC biofilm and TTSS.

## Methods

### Materials

Previously purified isolimonic acid, ichangin, isoobacunoic acid, IOAG and DNAG were used in the present study [36]. Purity of the individual limonoids was calculated from percent peak area using high performance liquid chromatography (HPLC) analysis [38]. A stock solution was prepared by dissolving 20 mg of each purified limonoid in 1 ml of dimethyl sulfoxide (DMSO).

### Bacterial strains and plasmids

Bacterial strains and plasmids used in the study are listed in Table 1. Unless otherwise specified, bacterial cultures were grown at 37°C in Luria-Bertani (LB) medium supplemented with 0.5% glucose. When appropriate, medium was supplemented with 10 μg of chloramphenicol or 100 μg of ampicillin per ml. Biofilm studies were carried out in colony forming antigen (CFA) medium [39,40]. Plasmids *pVS150* (*qseA* in *pACYC177*) and *pVS178* (*qseBC* in *pBAD33*) were purified from strains VS151 and VS179 respectively, using Qiagen Plasmid Purification Kit (Qiagen) and electroporated into EHEC ATCC 43895. The transformed strains were designated as AV43 (EHEC containing *pVS178*) and AV45 (EHEC containing *pVS150*). In addition, *pVS150* was electroporated into strain TEVS232 and resulting strain were designated as AV46. Furthermore, *qseB* and *qseC* were amplified from EHEC genomic DNA, using primers *qseB*_*(cloning)*_ and *qseC*_*(cloning)*_. The primers were designed by altering one base to create restriction sites for the respective enzymes. Amplified fragment of *qseC* was digested with SacI and SalI and cloned into *pBAD33*, generating plasmid *pAV11*. The *qseB* fragment was digested with SacI and HindIII and cloned into *pBAD33*, generating plasmid *pAV12*. Plasmids *pAV11* and *pAV12* were subsequently electroporated into EHEC ATCC 43895 and strains were designated as AV48 and AV49, respectively.

**Table 1:** Bacterial Strains used in the study

**Strain/Plasmid**	**Genotype**	**Reference/Source**
**Strains**		
*E. coli* O157:H7 EDL933	Wild type	ATCC (#43895)
TEVS232	*E. coli* TE2680 *LEE1:lacZ*	[41]
TEVS21	*E. coli* TE2680 *LEE2:lacZ*	[41]
VS145	EHEC 86–24 Δ*qseA*	[42]
VS151	VS145 with plasmid pVS150	[42]
VS138	EHEC 86–24 Δ*qseC*	[6]
VS179	VS138 with plasmid pVS178	[6]
AV43	WT with plasmid *pVS178*	This study
AV45	WT with *pVS150*	This study
AV46	TEVS232 with *pVS150*	This study
AV48	WT with *pAV11*	This study
AV49	WT with *pAV12*	This study
**Plasmids**		
*pVS150*	*qseA* into *pACYC177*	[42]
*pVS178*	*E. coli* K12 *qseBC* in *pBAD33*	[6]
*pAV11*	EHEC *qseC* in *pBAD33*	This Study
*pAV12*	EHEC *qseB* in *pBAD33*	This study
*pBAD33*	*pBAD33*	ATCC

### Growth and metabolic activity

The growth and metabolic activity of EHEC was measured as previously described [36]. Briefly, overnight cultures of EHEC were diluted 100 fold in LB media. Two hundred microliters of diluted cultures was placed in each well of 96-well plates and grown for 16 h at 37°C in presence of 6.25, 12.5, 50, or 100 μg/ml limonoids or equivalent volume of DMSO. The plates were constantly shaken at medium speed in Synergy™ HT Multi-Mode Microplate Reader (BioTek, Instruments, Winooski, VT). OD_600_ was recorded every 15 min. Metabolic activity of EHEC was measured by adding AlamarBlue (25 μl/well) and absorption at 570 and 600 nm was monitored in similar fashion as growth curve.

### Biofilm assay

EHEC biofilms were grown in polystyrene 96-well plates by plating 200 μl/well of 100 fold diluted overnight cultures in presence of 6.25, 12.5, 50, or 100 μg/ml of limonoids at 26°C for 24 h without shaking [23,39]. For VS138 (*ΔqseC*) and VS179 (VS138 + *qseBC*) biofilms were quantified after 48 h growth in 96-well plates. The biofilms were quantified by staining with 0.3% crystal violet (Fisher, Hanover Park, IL) for 20 min. Extra stain was washed with phosphate buffer (0.1 M, pH 7.4) and dye associated with attached biofilm was dissolved with DMSO. An absorbance at 570 nm was used to quantify the total biofilm mass.

### In vitro adhesion assay

Human epithelial Caco-2 cells were purchased from ATCC (Manassas, VA) and maintained in Dulbecco’s Minimal Essential Medium (DMEM) with nonessential amino acids and 10% fetal bovine serum without antibiotics. Caco-2 cells were seeded at 1 × 10^5^ cells/well in 6-well plates and infected with approximately 5 × 10^6^ cells/well of freshly grown EHEC ATCC 43895 in presence or absence of 100 μg/ml isolimonic acid, ichangin, isoobacunoic acid, IOAG and DNAG. The plates were incubated for 3 h at 37°C in 5% CO_2_ environment. After completion of incubation, plates were washed 3× with sterile PBS to remove any loosely attached cells. Caco-2 cells were lysed with 0.1% Triton-X in PBS to release the bacteria and serial dilutions were plated on LB-agar and incubated at 37°C for 24 h. Colonies were counted after incubation period and presented as log_10_CFU/ml.

### Caco-2 cell survival assay

Caco-2 cells (1 × 10^4^/well) were seeded in 96-well plate and exposed to 100 μg/ml of isolimonic acid, ichangin, isoobacunoic acid, IOAG and DNAG for 6 h in humidified incubator at 5% CO_2_, 37°C. Cell survival was determined by measuring lactate dehydrogenase using CytoTox-ONE™ Homogeneous Membrane Integrity Assay (Promega Corp., Madison, WI).

### Quantitative PCR

Relative transcript amount of selected genes (Table 2) was measured by qRT-PCR as described [23]. Briefly, overnight cultures of EHEC ATCC 43895 were diluted 100 fold with fresh LB medium or DMEM+10% FBS (referred as DMEM henceforth), treated with limonoids (100μg/ml) or DMSO and grown further at 37°C, 200 rpm. Bacterial cells were collected at OD_600_ ≈1.0. RNA was extracted using RNeasy minikit (Qiagen Inc., Valencia CA) and converted to cDNA using MuLV reverse transcriptase enzyme and random hexamer in a Reverse-Transcriptase polymerase chain reaction (RT-PCR) [43] at 42°C for 1 h. PCR products were purified with QIAquick PCR-purification kit (Qiagen Inc.). Twenty five nanogram cDNA from each sample was amplified with 10 pmol target primers using SYBR Green PCR master mix (Life Technologies Corporation, Carlsbad, CA) for 40 amplification cycles. After completion of 40 PCR cycles, melt curve data was generated. All the measurements were done on three biological replicates consisting of three technical replicates each. Amplification of target sequences was done on ABI-Prism 7000 HT (Applied Biosystems, Foster City, CA). The C_t_ values for primers were normalized against that of 16S rRNA. Fold change in the gene expression was calculated by 2^(−ΔΔCt)^[44] and expressed as fold change ±SD.

**Table 2:** Sequences of the Primers used in this study

**Primer**	**Sequence (5’-3’)**		**Reference**
	**Forward**	**Reverse**	
*cesD*	GTTTATCAAATCATGAAGATGCACAA	GCCCTGGGATCTTGCATAAC	[23]
*escJ*	CCAATGATGTCAATGTTTCCAAA	GCGCGAACAAAATCCTCTTT	[23]
*escR*	GCCAGCCTCCAACAAGAATG	ATTGGCCTTGGGTATGATGATG	[23]
*escU*	TCCACTTTGTATCTCGGAATGAAG	CAAGGATACTGATGGTAACCCTGAA	[23]
*flhC*	CGCTTTCCAGCATCTGCAA	CGGGATATTCAGCTGGCAAT	[23]
*flhD*	TCATTCAGCAAGCGTGTTGAG	TCCCGCGTTGACGATCTC	[23]
*ler*	CGACCAGGTCTGCCCTTCT	TCGCTCGCCGGAACTC	[23]
*sepZ*	CGGAGACGAGCAGCACAGA	CCGCCAACCGCAGTAAGA	[23]
*stx2*	ACCCCACCGGGCAGTT	GTCAAAACGCGCCTGATAGAC	[23]
*rpoA*	GTTGCCGCACGACGAATCGC	CCCAATCGGCCGTCTGCTGG	This study
*qseC*	CAGTCCACAGGGCAGCGTGG	AGTCCACTGCCGGTAGCGGT	This study
*qseB*	GAGCTGCGCCACGGTAACGT	AGTTTGCGCGGCAGTACCCG	This study
*qseA*	CCAGCCCCCGACCTGATTGC	GCGGGATCAGGCGAGTCGAG	This study
*qseB*_*(cloning)*_	GTGCTGTACAGAGCTCGTTACAAC	CCAGGCGACAAAGCTTGAAAGCA	This study
*qseC*_*(cloning)*_	TGCGTCTGGGAGCTCACGATTATC	GGTGAGACGTTTGTCGACTATAGTACG	This study

The underlined segment in AV25/26 and AV29/30 indicate the restriction enzyme sites.

### AI-3 reporter assay

Preconditioned media (PM) was prepared as described [41]. Overnight cultures of TEVS232, TEVS21 and AV45 (EHEC ATCC 43895 harboring *pVS150*) were diluted 100 fold in LB medium and grown till OD600 ≈0.2. The cells were collected by centrifugation at 2500 × g for 10 min and resuspended in either fresh LB media supplemented with 50 μM epinephrine or PM and treated with 100 μg/ml isolimonic acid or equivalent amount of DMSO. The β-galactosidase activity was measured after 30 min incubation at 37°C using *o*-nitrophenyl β-D-galactopyranoside as previously described [45] and reported as mean ± SD of three replicates.

### Statistical analysis

Percent inhibition of biofilm formation was calculated from three experiments consisting of three replicate wells using the formula 100- [(OD_570_ of sample well/ OD_570_ of positive control) × 100]. Effects of different limonoids for each activity were analyzed using analysis of variance (ANOVA) followed by Tukey’s pairwise multiple comparison test on SPSS 16.0 (SPSS Inc., Chicago, IL, USA). The effect was considered significant at p <0.05. The data for EHEC biofilm was fitted to a 3-parameter sigmoid models y= a/(1+exp(−(x-x0)/b)) using SIGMAPLOT 11.0 (Systat Software, Inc.). In order to conduct the analysis, concentration of each limonoids was converted to Log_10_ μM and plotted against percent inhibition values.

## Results

### Effect of citrus limonoids on EHEC growth and biofilm formation

The purity of all tested limonoids was >95% (Figure 1). Furthermore, limonoids in the concentration range of 6.25-100 μg/ml, did not affect EHEC growth (Table 3) and viability (Additional file 1: Figure S1).

**Table 3:** **Generation time (in minutes) of *E. coli *O157:H7 upon exposure of different concentrations of limonoids**

**Concentration (μg/ml)**	**DMSO**	**IL**	**IBA**	**Ichangin**	**DNAG**	**IOAG**
100	23.56 ± 0.71	23.11 ± 0.76	22.97 ± 0.96	23.65 ± 0.95	23.58 ± 1.06	22.96 ± 1.06
50	24.90 ± 1.82	22.97 ± 0.97	23.12 ± 0.92	23.16 ± 0.93	23.27 ± 1.09	23.64 ± 1.08
25	23.62 ± 2.47	23.58 ± 1.19	23.26 ± 1.23	22.58 ± 1.26	23.68 ± 0.91	23.51 ± 1.26
12.5	23.68 ± 1.84	23.54 ± 1.01	22.69 ± 1.09	23.12 ± 1.08	23.97 ± 1.31	23.69 ± 1.32
6.25	23.91 ± 0.63	23.70 ± 1.09	23.90 ± 1.02	23.55 ± 1.05	23.61 ± 1.05	23.76 ± 1.01

The mean ± SD of three replicates are presented.

**Figure 2:**
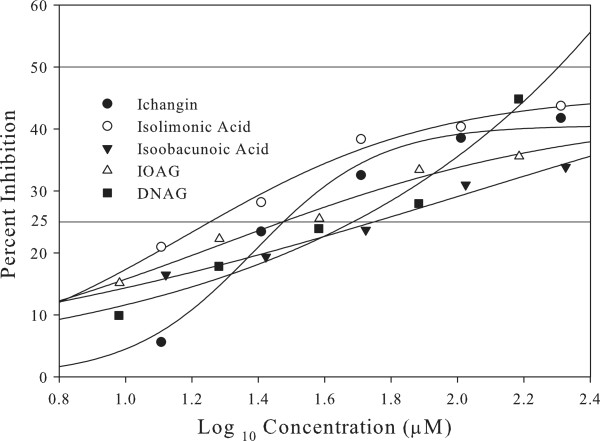
**Three parameter models of biofilm formation inhibition by citrus limonoids.** Line curves at 50% and 25% represent the IC_50_ and IC_25_ values for compounds. Biofilms were grown in 96-well plates and quantified using crystal violet. Percent inhibition over solvent control (DMSO) was calculated. To generate 3-parameter models, concentrations were changed to Log10 μM and plotted against percent inhibition.

All the five limonoids inhibit biofilm formation in concentration dependent manner (Figure 2). Biofilm inhibitory activities of limonoids were compared by calculating IC_25_ values from 3-parameter sigmoid equations (Figure 2). The 3-parameter equation was chosen due to better fit demonstrated for 4 out of 5 limonoids. IC_25_ values were used for comparison because limonoids demonstrated <50% inhibition of biofilm formation. The R^2^ values for isolimonic acid, ichangin, isoobacunoic acid, IOAG and DNAG were 0.99, 0.96, 0.92, 0.88 and 0.99 respectively. Isolimonic acid was the most potent inhibitor of biofilm formation among the tested limonoids with an IC_25_ of 19.7 μM (Figure 2) followed by ichangin (IC_25_ = 28.3 μM). IOAG was more potent (IC_25_= 29.54 μM) than its aglycone isoobacunoic acid (IC_25_= 57.2 μM). Furthermore, 95% confidence intervals for IC_25_ values were calculated as 8.9-27.1 μM (isolimonic acid), 20.3-38.7 μM (ichangin), 17.9-54.6 μM (IOAG), 43.0-71.5 μM (isoobacunoic acid) and 23.0-66.1 μ M (DNAG).

### Effect of limonoids on adhesion of EHEC to Caco-2 cells

**Figure 3:**
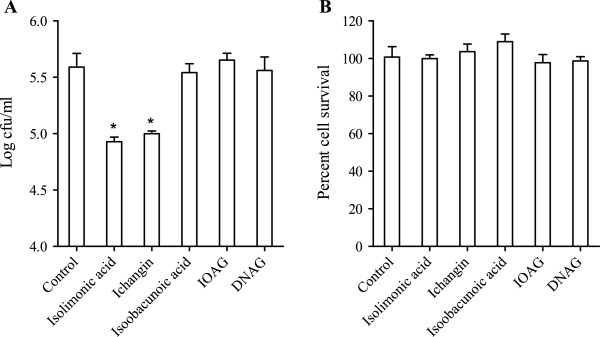
**Effect of limonoids on EHEC adhesion and survival of Caco-2 cells.** (**A**) Adhesion of EHEC to Caco-2 cells. Caco-2 cells were infected with 50 fold EHEC ATCC 43895 for 3 h. The EHEC cell numbers were enumerated by lysing the Caco-2 cells and plating the lysate on LB-agar plates, followed by counting colonies after 24 h. The data represents mean of three biological replicates and SD. Asterisk denotes significant (p<0.05) difference from solvent control (DMSO). (**B**) Survival of Caco-2 cells in presence of 100 μg/ml limonoids. The cell viability was measured by LDH assay after 6 h of growth in presence of limonoids.

To further understand the effect of limonoids, adherence of EHEC to colon epithelial Caco-2 cells was studied. Isolimonic acid and ichangin (100 μg/ml) treatment significantly (p<0.05) reduced the number of EHEC cells attached to Caco-2 cells by 0.66 and 0.59 Log_10_ cfu/ml, respectively (Figure 3A). Isoobacunoic acid, IOAG and DNAG did not affect the number of EHEC cells adhering to Caco-2 cells. To determine, if the observed reduction in adhesion of EHEC was due to reduced cell viability of Caco-2 cells, survival of Caco-2 in presence of 100 μg/ml limonoids at 6 h was assayed by measuring extracellular LDH. Survival of Caco-2 cells in presence of 100 μg/ml limonoids was similar to solvent control (Figure 3B).

### Citrus limonoids repress the LEE, flagellar and *stx2* genes

Adherence of EHEC to epithelial cells is facilitated by several factors including locus of enterocyte effacement (LEE) encoded TTSS, flagella, effector proteins and intimin [46-48]. To determine the probable mode of action, effect of limonoids on expression of six LEE encoded genes *ler*, *escU*, *escR* (LEE1 encoded), *escJ*, *sepZ* and *cesD* (LEE2 encoded), flagellar master regulators *flhDC* and *stx2* was studied. Isolimonic acid and ichangin exerted the strongest effect on the LEE in EHEC grown to OD_600_ ≈ 1.0 in LB media. The transcriptional regulator of LEE, the *ler*, was repressed 5 fold by isolimonic acid, while other LEE encoded genes were down-regulated by 6–10 fold (Table 4). Ichangin treatment resulted in ≈ 2.5-6 fold repression of LEE encoded genes. IOAG repressed the *escU*, *escR*, *escJ* and *cesD* by 3.2, 2.5, 3.7 and 2.6 fold, respectively while aglycone, isoobacunoic acid did not seem to affect the expression of LEE encoded genes under investigation (Table 4). Similarly, DNAG treatment did not resulted in differential expression of any genes. Furthermore, isolimonic acid repressed the *flhC* and *flhD* by 4.5 and 6.9 fold, respectively (Table 4), while ichangin exposure resulted in 2.8 fold repression of *flhC* and *flhD*. IOAG repressed *flhC* and *flhD* by 2.1 and 2.3 folds, respectively. Isoobacunoic acid and DNAG treatment did not seem to modulate the expression of *flhDC* (Table 4).

**Table 4:** Expression of LEE encoded, flagellar and stx2 genes in presence of 100 μg/ml limonoids

**Gene name**	**Ichangin**	**Isolimonic acid**	**Isoobacunoic acid**	**IOAG**	**DNAG**
*ler*	-3.2 (±2.1)	-5.0 (±0.8)	-1.4 (±0.3)	-1.8 (±0.4)	-0.7 (±1.5)
*escU*	-5.3 (±0.8)	-6.6 (±1.0)	-1.6 (±0.1)	-3.2 (±0.3)	-2.0 (±0.6)
*escR*	-2.5 (±0.7)	-6.3 (±1.3)	-1.7 (±0.3)	-2.5 (±1.2)	-2.3 (±0.5)
*escJ*	-6.2 (±1.0)	-12.4 (±2.1)	-2.4 (±1.3)	-3.7 (±2.0)	-1.2 (±2.4)
*sepZ*	-2.7 (±0.1)	-6.9 (±1.1)	-0.7 (±1.5)	-1.7 (±0.6)	-1.6 (±0.8)
*cesD*	-3.5 (±0.7)	-10.0 (±1.5)	-3.0 (±1.2)	-2.6 (±1.7)	-1.6 (±0.8)
*flhC*	-2.8 (±0.9)	-4.5 (±1.3)	-1.5 (±0.3)	-2.1 (±0.4)	-1.3 (±0.3)
*flhD*	-2.8 (±0.5)	-6.9 (±0.4)	-1.8 (±0.5)	-2.3 (±0.4)	-1.7 (±0.5)
*stx2*	-2.5 (±0.8)	-4.9 (±1.0)	-1.6 (±0.4)	-2.2 (±0.8)	-1.2 (±0.1)
*rpoA*	-0.3 (±1.8)	-0.5 (±1.6)	1.8 (±0.8)	1.3 (±0.4)	1.7 (±0.5)

The EHEC ATCC 43895 was grown to OD600≈1.0, RNA was extracted using RNeasy kit and converted to cDNA as described in text. Target genes were amplified from three biological samples. Fold change was calculated using 2^(−ΔΔCt)^ method and presented as mean ± SD of three replicates.

Shiga toxin produced by EHEC is responsible for HUS [2]. We were further interested in learning if any of the limonoids modulate expression of *stx2*. Isolimonic acid and ichangin (100 μg/ml) repressed the *stx2* by 4.9 and 2.5 fold, respectively (Table 4), while IOAG, isoobacunoic acid and DNAG did not seem to affect the expression of *stx2*.

**Figure 4:**
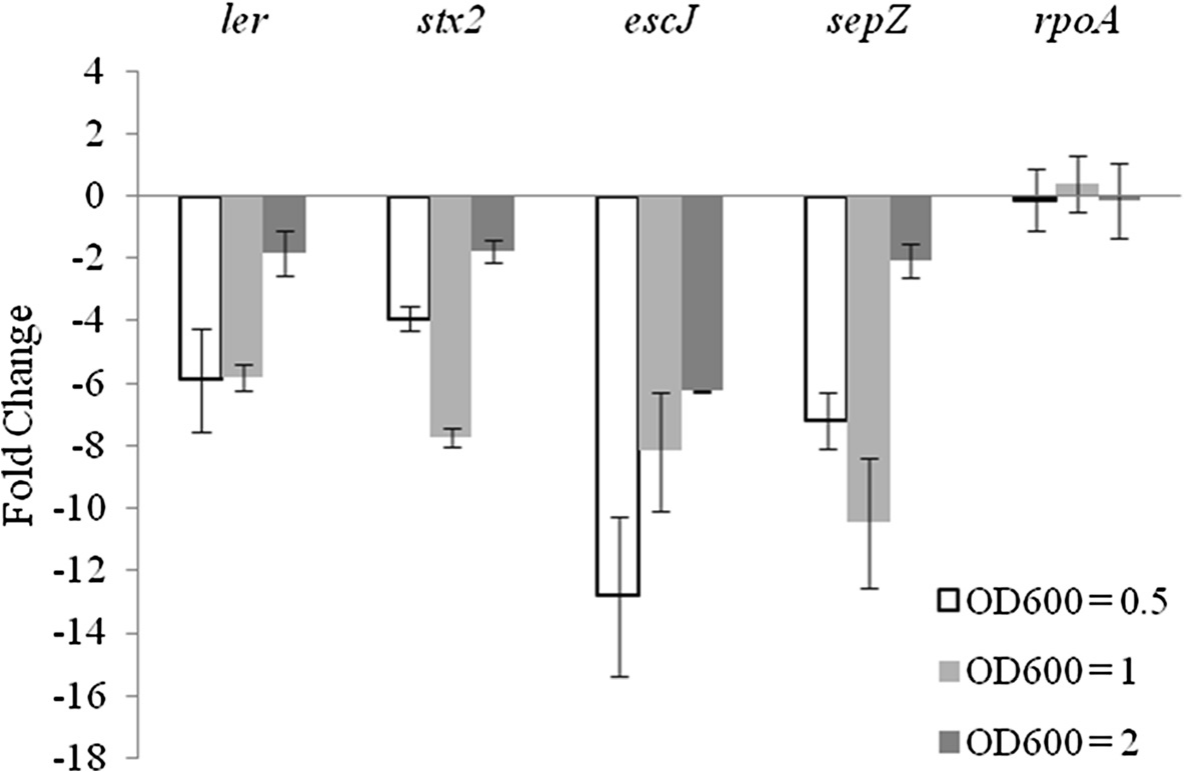
**Expression of LEE encoded genes in DMEM in response to isolimonic acid.** Fold change in expression were calculated as isolimonic acid over DMSO. The data represents mean of three biological replicates and SD. The samples were collected at OD_600_ of 0.5, 1.0 and 2.0 and processed as described in Materials and Methods.

The culture of EHEC in DMEM was reported to activate LEE expression [41]. To determine, if isolimonic acid represses LEE under DMEM growth conditions, expression of *ler*, *stx2*, *escJ* and *sepZ* were measured. Isolimonic acid treatment repressed *ler*, *stx2*, *escJ* and *sepZ* in DMEM media by >5, 7, 8 and 10 fold whereas, expression of *rpoA* was unaffected (Figure 4). The *escJ* and *sepZ*, which are coded as a polycistronic message, demonstrated differing levels of regulation in presence of isolimonic acid (Figure 4). However, differential degradation and processing of genes encoded as polycistronic mRNA is well documented [49,50], and could potentially be the reason of different levels of mRNA transcripts recorded for *escJ* and *sepZ*.

### Effect of isolimonic acid on AI-3/epinephrine induced LEE expression

**Figure 5:**
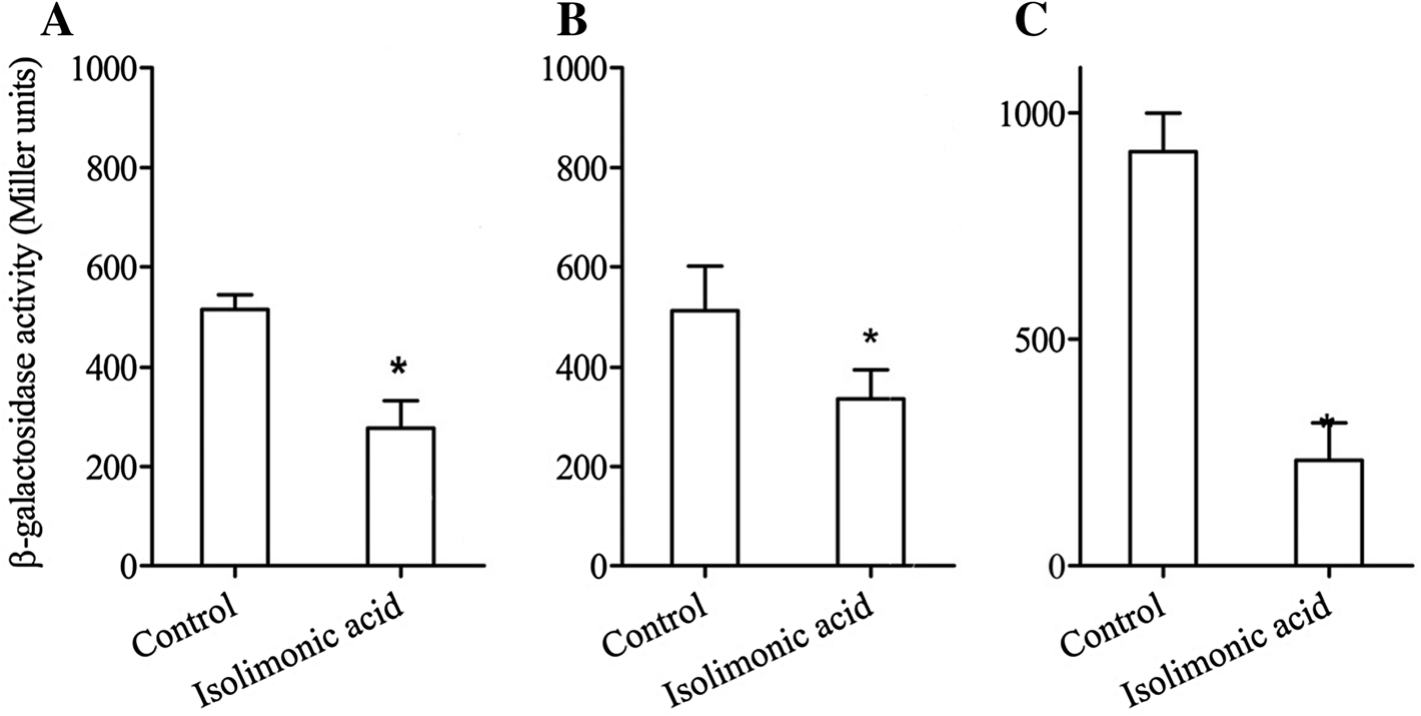
**Effect of isolimonic acid on AI-3/epinephrine mediated signaling.** Inhibition of preconditioned media induced β-galactosidase activity in (**A**) TEVS232 (LEE1) and (**B**) TEVS21 (LEE2) by 100 μg/ml isolimonic acid or DMSO (control). Preconditioned media was prepared as described in text. (**C**) Epinephrine induced β-galactosidase activity in TEVS232 in presence of 100 μg/ml isolimonic acid or solvent control (DMSO). The EHEC was grown to OD600 ≈ 0.2, collected by centrifugation and resuspended in preconditioned medium or media supplemented with 50 μM epinephrine. Isolimonic acid or DMSO were added and β-galactosidase activity was measured after 30 min incubation. Asterisk denotes significant (p<0.05) difference from solvent control (DMSO).

AI-3/epinephrine mediated cell-cell signaling regulates biofilm, motility and expression of LEE in EHEC [6,12,15]. To ascertain if isolimonic acid interferes with AI-3 signaling, reporter strains TEVS232 and TEVS21 were induced by PM in presence of 100 μg/ml isolimonic acid, and β-galactosidase activity was measured. TEVS232 and TEVS21 contain single copy operon fusions of *LEE1:LacZ* and *LEE2:LacZ*, respectively [41]. Isolimonic acid treatment reduced the expression of *LEE1* (TEVS232) and *LEE2* (TEVS21) by 46.05 and 34.23%, respectively (Figure 5A and B). Additionally, *LEE1* was stimulated by 50 μM epinephrine in presence or absence of 100 μg/ml isolimonic acid and β-galactosidase activity was measured. Isolimonic acid repressed the epinephrine-induced expression of *LEE1* by ≈3.9 fold (74.42 % reduction) (Figure 5C).

### QseBC dependent inhibition of biofilm by isolimonic acid

**Figure 6:**
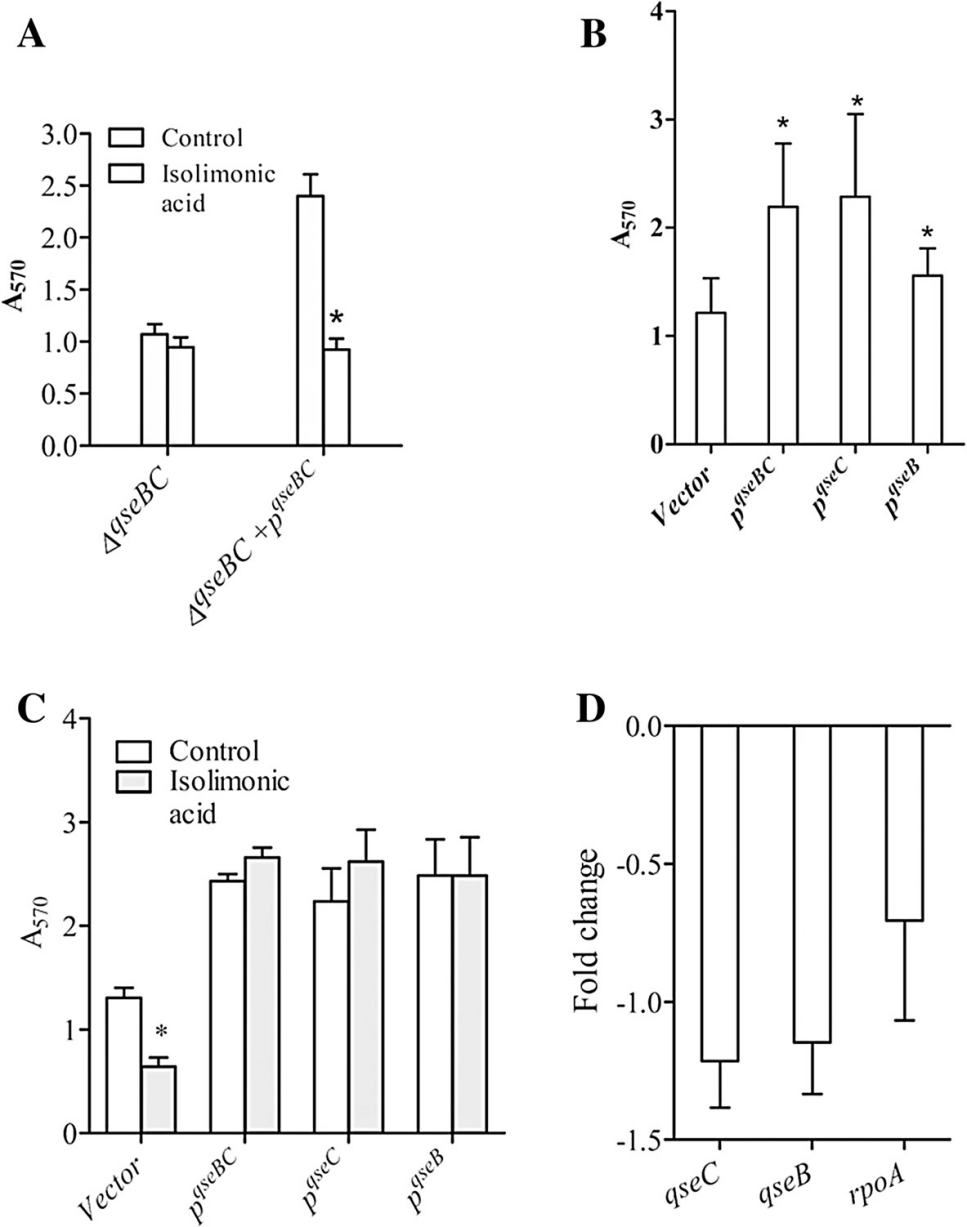
**Activity of isolimonic acid is dependent on QseBC.** Inhibition of biofilm in (**A**) *ΔqseBC* mutant and *ΔqseBC* mutant complemented with *qseBC* (*pVS178*). (**B**) Biofilm formation in EHEC supplemented with *qseBC*, *qseB* and *qseC*. Asterisk denotes significant (p<0.05) difference from vector control. (**C**) Inhibition of biofilm by 100 μg/ml isolimonic acid in EHEC supplemented with *qseBC*, *qseB* and *qseC*. Asterisk denotes significant (p<0.05) difference from solvent control (DMSO). (**D**) Expression of *qseB* and *qseC* in presence of 100 μg/ml isolimonic acid. The fold changes in expression were calculated as isolimonic acid over DMSO. The experiments were conducted in triplicate and mean ± SD are presented.

QseBC is a two component system, which detects AI-3 and epinephrine and modulates biofilm formation and flagellar expression [6]. As isolimonic acid seems to interfere with AI-3/epinephrine induced pathway, it was possible that this interference is dependent on QseBC. To determine if isolimonic acid inhibits EHEC biofilm formation by affecting QseBC, biofilm formation in EHEC 86–24, QseC deletion mutant (VS138) and complemented strain VS179 [6] was studied. Since *ΔqseBC* strain (VS138) did not form appreciable biofilm at 24 h, the biofilms were grown up to 48 h. The biofilm formation in *ΔqseBC* at 48 h was similar between solvent control (DMSO) and isolimonic acid (p>0.05) (Figure 6A). In contrast, isolimonic acid reduced the biofilm formation by 61.33% in complemented strain VS179. To further understand the role of QseBC in wild type strain ATCC 43895, plasmid *pVS178* (carrying *qseBC*), was purified from VS179 and introduced into wild type strain. In addition, *qseB* and *qseC* were amplified from EHEC genomic DNA, cloned into *pBAD33* vector and introduced into EHEC strain ATCC 43895. The expression of *qseBC*/*qseB*/*qseC* was induced by addition of 0.2% arabinose in the media. Overexpression of *qseBC/qseC/qseB* formed significantly more biofilm, when compared to EHEC wild type carrying vector alone (Figure 6B). We further measured the effect of isolimonic acid on the biofilm formation in strains overexpressing *qseBC/qseC/qseB* (Figure 6C). The isolimonic acid treatment did not significantly affect the biofilm formation, measured after 24 h of growth, in EHEC strains overexpressing *qseBC*/*qseC*/*qseB* (Figure 6C). Furthermore, it was possible that isolimonic acid modulates the expression of *qseBC* leading to inhibition of biofilm. To determine the effect of isolimonic acid, expression of *qseB* and *qseC* was measured by qRT-PCR. The results indicate that isolimonic acid do not regulate the expression of *qseB* and *qseC* (Figure 6C). Altogether, finding of these experiments seem to suggest that isolimonic acid affects the QseBC activity but not the expression to inhibit biofilm formation.

### QseA dependent inhibition of *ler* by isolimonic acid

**Figure 7:**
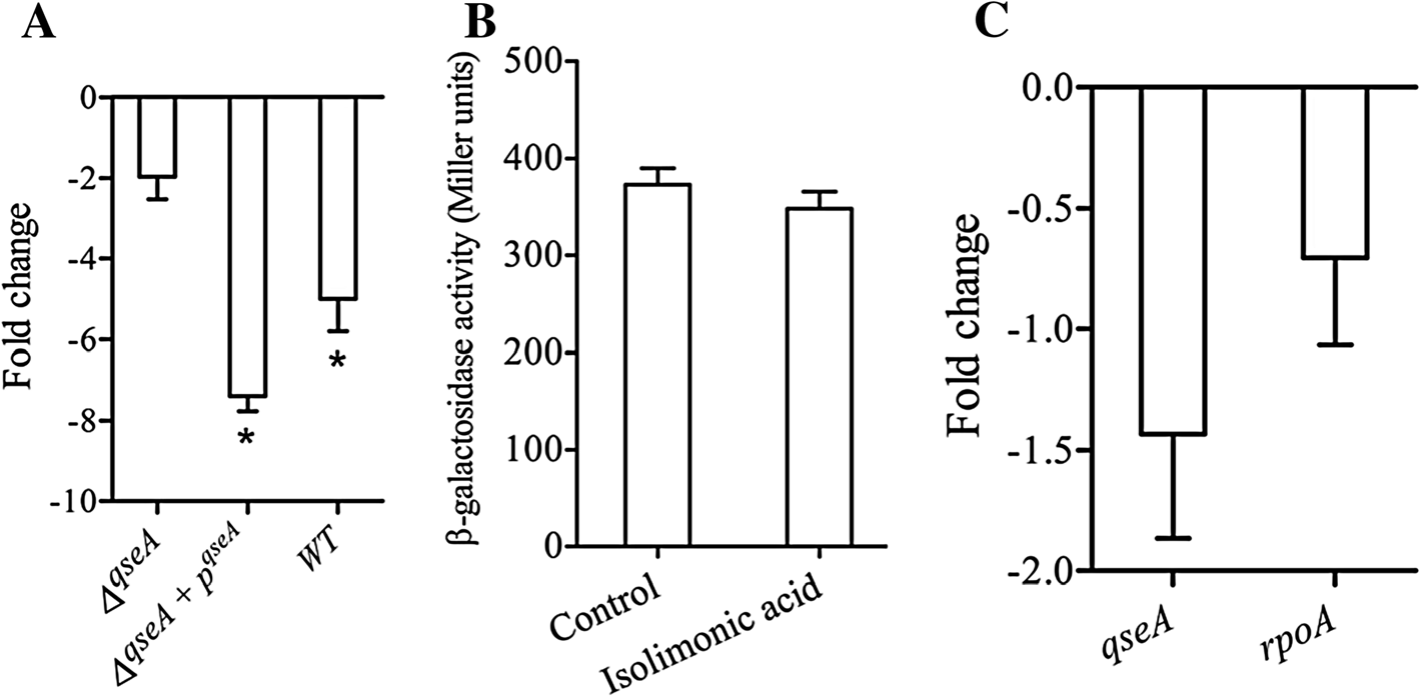
**Isolimonic acid requires QseA to repress ler.** (**A**) Expression of *ler* in *ΔqseA* mutant and *ΔqseA* mutant supplemented with *p*^*qseA. *^The expression was monitored 30 min after addition of preconditioned media and 100 μg/ml isolimonic acid. (**B**) AI-3 induced β-galactosidase activity in TEVS232 supplemented with *qseA* (AV46). Asterisk denotes significant (p<0.05) difference from solvent control (DMSO). (**C**) Expression of *qseA* in presence of 100 μg/ml isolimonic acid. Fold change values were calculated over EHEC grown in presence of DMSO. The data represents mean ±SD of triplicate experiment.

Repression of LEE and interference of AI-3/epinephrine mediated signaling by isolimonic acid prompted us to investigate the role of QseA. To determine the contribution of QseA, change in *ler* expression was monitored in *qseA* deletion (VS145) and complemented (VS151) strains. Isolimonic acid (100 μg/ml) treated cultures demonstrated a <2 fold change in *ler* expression in *qseA* deletion mutant. In comparison, isolimonic acid repressed the *ler* by 7.4 fold in complemented strain VS151 (Figure 7A). To further confirm the role of QseA, *qseA* was overexpressed by introducing the plasmid *pVS150*, harboring *qseA*, into reporter strain TEVS232 and expression of chromosomal fusion *LEE1:LacZ* (β-galactosidase activity) was measured. Overexpression of *qseA* from a multicopy plasmid negated the inhibitory activity of isolimonic acid (Figure 7B). Furthermore, the possibility of transcriptional regulation of *qseA* by isolimonic acid was determined by assessing the *qseA* expression. A < 2 fold change in the transcript levels of *qseA* indicated that isolimonic acid do not regulate the expression of *qseA* (Figure 7C). Altogether, the isolimonic acid appears to repress *ler* expression and possibly LEE by modulating QseA activity.

## Discussion

EHEC is an important gastrointestinal pathogen, prolific biofilm former and demonstrates resistance to various antimicrobials in biofilm mode of growth [51]. For successful colonization of gastrointestinal tract and initiation of infection, adhesion of EHEC to intestinal epithelium is an essential early event [47,48]. Additionally, several *E. coli* pathovars were reported to produce and live in biofilms inside the human body [19]. In order to counteract these maladies, an antivirulence molecule with anti-adhesion and/or anti-biofilm properties may be highly desirable. Research in our laboratory has identified several molecules with differing anti-virulence effects [23,28,36,37,52,53]. The current work examined the potential of five citrus limonoids- isolimonic acid, ichangin, isoobacunoic acid, IOAG and DNAG, to inhibit EHEC biofilm and TTSS. All the tested limonoids seem to interfere with the EHEC biofilm formation in a dose dependent fashion (Figure 2). Isolimonic acid was the most potent inhibitor of the EHEC biofilm and adhesion to Caco-2 cells. Moreover, the limonoids do not seem to affect growth of EHEC, suggesting that limonoids, especially isolimonic acid inhibits EHEC biofilm and adhesion without adversely affecting the growth or metabolic activity (Table 1, Additional file 1: Figure S1).

In EHEC, the initial attachment to various surfaces such as epithelial cells and plastic surface is regulated by several factors including TTSS, flagella and fimbriae [47,48,54]. LEE encoded TTSS, effector proteins as well as flagella and intimin [47,48] play an important role in adhesion of EHEC to gastrointestinal tract surface, while flagella and fimbriae also contribute in biofilm formation. Results of the adhesion and biofilm assay indicated that one or more of above-mentioned factors may be affected by limonoids particularly by isolimonic acid. To investigate this hypothesis, expression of LEE encoded genes and flagellar master regulators *flhDC* was determined by qRT-PCR. Isolimonic acid and ichangin appear to exert their antivirulence and biofilm inhibitory effect by repressing TTSS carried on LEE, *stx2*, which encodes for Shiga toxin and flagellar master regulon *flhDC* (Table 4).

In EHEC, expression of LEE and flagellar operons are regulated by multiple environmental and genetic factors including QS [10-13]. In particular AI-2/AI-3/epinephrine mediated cell-cell signaling regulates the expression of both flagellar operon and LEE, which contribute to adhesion and biofilm formation. Furthermore, expression of *stx2* is also regulated by QS [2,12,55,56]. Therefore, repression of TTSS, flagella and *stx2* indicated a possibility that limonoids, especially isolimonic acid may interfere with EHEC QS. Isolimonic acid was chosen for further studies, as it demonstrated the most potent inhibition of biofilm formation, adhesion, LEE, *flhDC* and *stx2*. For determination of AI-3/epinephrine mediated QS in EHEC, reporter strains TEVS 232 and TEVS21 containing chromosomal fusions *LEE1:LacZ* and *LEE2:LacZ* were used. The analysis was confined to *LEE1* and *LEE2*, because these two operons have been reported to be directly activated by AI-3/epinephrine mediated QS [15,41]. To test if the isolimonic acid acts as an QS inhibitor, PM/epinephrine stimulated activation of *LEE1* and *LEE2* in reporter strains was measured [41]. The PM, described earlier [41], was used as a source of AI-3 molecules as the purified AI-3 was not available. Repression of AI-3/epinephrine-induced *ler*, *LEE1* and *LEE2* (Figure 5) indicated that isolimonic acid interferes with EHEC QS system.

The autoinducers and hormones reportedly increase the autophosphorylation levels of histidine kinase QseC, which then activates QseB to regulate motility and biofilm formation [57]. Furthermore, interaction of AI-3/epinephrine with QseA activates LEE encoded genes [15,57]. It was possible that isolimonic acid interferes with EHEC QS in a mechanism involving QseBC and QseA. If activity of isolimonic acid depends upon functional QseBC, deletion of *qseBC* will eliminate the inhibitory effect. On the other hand, complementation of *ΔqseBC* with plasmid borne QseBC is likely to restore the inhibitory effect of isolimonic acid. Furthermore, overexpression of *qseBC* in wild type background (EHEC ATCC 43895) will result in higher levels of QseBC proteins in the cell and consequently will have a higher activity. This higher level of activity may compensate and relieve the inhibitory effect of isolimonic acid on biofilm formation. In order to verify QseBC dependent inhibition, biofilm formation in *ΔqseBC* strain (VS138) and complemented strain (VS179) [6] in presence of 100 μg/ml of isolimonic acid was measured. As expected, isolimonic acid did not reduce the biofilm formation in VS138. In contrast, isolimonic acid exposure resulted in a significant decrease in VS179 (*qseBC* complemented strain) biofilm as measured by crystal violet (Figure 6A), indicating involvement of QseBC. Additionally, overexpression of *qseBC*, *qseB* and *qseC* in EHEC ATCC 43895, under the control of arabinose operon restored the inhibitory effect of isolimonic acid on EHEC biofilm formation (Figure 6B). Taken together these results suggest that effect of isolimonic acid is dependent upon QseBC. Furthermore, the effects of isolimonic acid did not seem to arise from modulation of *qseBC* expression. However, based on the current data it was not possible to differentiate, if the effect is dependent solely upon *qseB* or *qseC*, as supplementation of EHEC by both *qseB* and *qseC* relieved the inhibitory effect. Further studies are required to precisely determine if the target of isolimonic acid is *qseB* or *qseC*.

**Figure 8:**
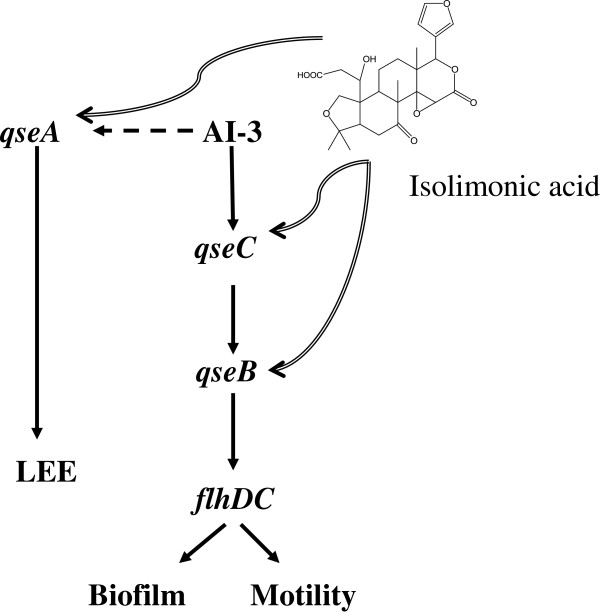
**Hypothetical model of isolimonic action on EHEC.** The isolimonic acid seems to modulate the AI-3/Epinephrine mediated signaling in QseBC and QseA dependent manner. Broken arrow indicate unknown mode of interaction of AI-3 with *qseA*. Wavy arrows indicate interaction of isolimonic acid with *qseBC* and *qseA* is unknown.

To understand the role of QseA in isolimonic acid mediated repression of LEE, expression levels of transcriptional regulator *ler* were measured as QseA is reported to directly activate expression of *ler*[15]. Ler is the transcriptional regulator of the genes encoded in LEE and activates the genes encoded in LEE [15,21]. We hypothesized that if isolimonic acid affect *ler* via QseA, the *ler* expression will not change in *ΔqseA* strain (VS145) but complementation of *qseA* (strain VS151) from plasmid will restore the inhibitory effect. In addition, overexpression of *qseA* in wild type strain ATCC 43895 will negate the inhibitory effect of isolimonic acid. The hypothesis was tested by measuring the expression of *ler* using qRT-PCR in VS145 and VS151, grown in presence of 100 μg/ml isolimonic acid and compared with DMSO. The results demonstrated that expression of *ler* was not significantly altered in *ΔqseA* strain (VS145), whereas a 7.4 fold repression of *ler* (Figure 7A) was observed in *qseA* complemented strain (VS179). Furthermore, overexpression of *qseA* from multicopy plasmid *pVS150* in TEVS232 background (AV46) nullified the repressive effect (Figure 7B) of isolimonic acid on *LEE1* observed in Figure 5A. Collectively the data indicated that repression of LEE by isolimonic acid is dependent on QseA. However, isolimonic acid does not seem to transcriptionally modulate the expression of *qseA*. Thus the results of the study indicate towards a model where isolimonic acid modulates the biofilm and TTSS in QseBC and QseA dependent fashion, however without regulating the expression of these genes (Figure 8).

## Conclusion

The present study demonstrates that the citrus limonoids, particularly isolimonic acid and ichangin are strong inhibitors of biofilm formation and attachment of EHEC to Caco-2 cells. Furthermore, isolimonic acid and ichangin seems to affect biofilm formation and TTSS by repressing LEE and flagellar operon. Isolimonic acid seems to exert its effect by inhibiting AI-3/epinephrine mediated cell-cell signaling in QseBC and QseA dependent manner. However, the mechanism by which isolimonic acid affects the QseBC and QseA remains to be elucidated. One possibility is that the isolimonic acid may interfere with the DNA binding activities of QseB and QseA. Another possible scenario will be that isolimonic acid interferes with phosphorylation events. However, further study is required to determine the target of isolimonic acid for the modulation of *flhDC* and *ler*. In addition, determination of the structure-activity relationship will provide further insights into the inhibitory action of isolimonic acid. In nutshell, isolimonic acid acts as an antivirulence agent in EHEC and may serve as lead compound for development of novel agents. Furthermore, the fact that isolimonic acid is present in citrus juices and do not demonstrate cytotoxic effect on normal human cell line [58], increases the desirability to develop it as antivirulence agent.

## Competing interests

The authors declare that they have no competing interests.

## Authors’ contributions

AV, PRJ, SDP and BSP designed the study. AV performed the experiments. SDP and BSP supervised the study. AV and PRJ wrote the manuscript. All authors read and approved the final manuscript.

## Supplementary Material

Additional file 1: Figure S1 Metabolic activity of E. coli O157:H7 in presence of 100 μg/ml limonoids as measured by AlamarBlue reduction. Click here for file
