# Supplementary material for: Isolimonic acid interferes with Escherichia coli O157:H7 biofilm and TTSS in QseBC and QseA dependent fashion
Source: BMC Microbiol. 2012 Nov 15;12:261. doi: 10.1186/1471-2180-12-261 (PMC3562146; doi:10.1186/1471-2180-12-261)

FIG. S1: Metabolic activity of E. coli O157:H7 in presence of 100 µg/ml limonoids as measured by AlamarBlue reduction.


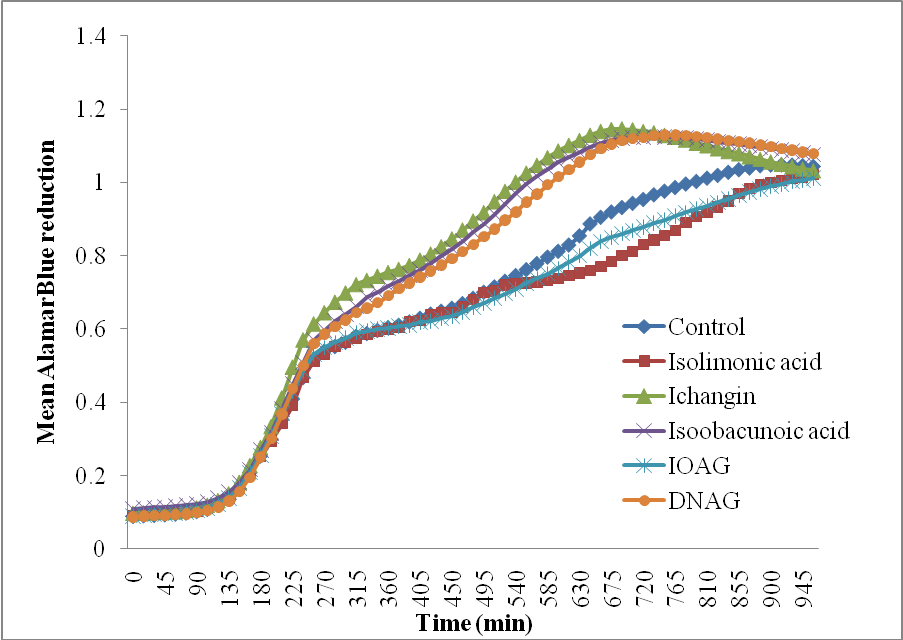

Supplement: Additional file 1: Figure S1 — Metabolic activity of E. coli O157:H7 in presence of 100 μg/ml limonoids as measured by AlamarBlue reduction. [file 1471-2180-12-261-S1.doc]
